# TKI or TKI combined with PD-1 inhibitors as second-line treatment for HCC patients after sorafenib failure

**DOI:** 10.3389/fphar.2022.1026337

**Published:** 2022-12-09

**Authors:** Jin Lei, Bowen Chen, Meiru Song, Linzhi Zhang, Xinfeng Zhang, Xiaoqiang Gao, Yinyin Li, Yinying Lu, Shi Zuo

**Affiliations:** ^1^ Guizhou Medical University, Guiyang, China; ^2^ Peking University 302 Clinical Medical School, Beijing, China; ^3^ The Fifth Medical Center of PLA General Hospital, The Fifth School of Clinical Medicine, Anhui Medical University, Hefei, China; ^4^ Comprehensive Liver Cancer Center, The Fifth Medical Center of the PLA General Hospita, Beijing, China; ^5^ Department of Hepatobiliary Surgery, Affiliated Hospital of Guizhou Medical University, Guiyang, China; ^6^ Guangdong Key Laboratory of Epigenetics, College of Life Sciences and Oceanography, Shenzhen University, Shenzhen, China

**Keywords:** hepatocellular carcinoma, tyrosine kinase inhibitor (EGFR-TKI), programmed death-1 inhibitor, second-line, sorafenib

## Abstract

**Background:** Tyrosine kinase inhibitors (TKI) in combination with programmed cell death-1 (PD-1) inhibitors become the potential treatment modality for patients undergoing unresectable hepatocellular carcinoma (uHCC) in the first-line setting. However, the efficacy and safety of this combination regimen in patients after sorafenib failure remains unclear.

**Methods:** Participants in this study included patients with uHCC after sorafenib failure who received TKI monotherapy (TKI group) or TKI combined with PD-1 inhibitors therapy (combination group) in our center from July 2018 to July 2021. The overall survival (OS) was used to be the primary efficacy endpoint, while progression-free survival (PFS), objective response rate (ORR), and disease control rate (DCR) were applied to be secondary endpoints. In addition, the adverse events are recorded and evaluated.

**Results:** Among the 92 patients contained in this work, 50 patients were categorized into the TKI group, while 42 patients were in the combination group. There existed no evident differences between the two groups concerning the ORR (8.0% vs. 9.5%, *p* = 1.000). However, the DCR in the combined group was better in relative to that in the TKI group (71.4% vs. 50.0%, *p* = 0.037). In comparison with the TKI group, it was found that the combination group presented notably better median PFS (8.1 months vs. 4.7 months, *p* = 0.005) and median OS (21.9 months vs. 16.6 months, *p* = 0.042). According to multivariate analysis, PFS (HR 0.5, 95% CI: 0.3–0.8, *p* = 0.005) and OS (HR 0.5, 95% CI: 0.3–1.0, *p* = 0.051) were improved in the combination group in relative to the TKI group after the adjustment for some risk factors. Additionally, the incidence rates of grade ≥1 adverse event in the TKI group and the combination group were 96.0% and 97.6%, respectively. The most normal adverse event in the TKI group was neutropenia (*n* = 24,48.0%) and the combination group was hypoalbuminemia (*n* = 23,54.8%). All of these adverse events improved after symptomatic treatment, and no new toxic events were found to occur.

**Conclusion:** TKI combined with PD-1 inhibitors showed better prognosis with manageable toxicity in uHCC patients after sorafenib failure compared with TKI monotherapy.

## Introduction

According to the latest statistics, primary liver cancer ranks the sixth most normal cancer type globally, with more than 900,000 new cases every year (Sung et al., 2021). Hepatocellular carcinoma (HCC) is found to occupy 85%–90% among all the primary liver cancers (El-Serag and Rudolph, 2007). Due to the early asymptomatic and rapid progress, most patients were diagnosed with advanced-stage disease. Patients who lost the opportunity of local therapy could only choose the best supportive treatment until the emergence of sorafenib brought them hope in 2007 (Llovet et al., 2008). The first-line treatment drugs approved by the Food and Drug Administration (FDA), from sorafenib in 2007 to lenvatinib with non-inferior effect to sorafenib in 2018, and then to atezolizumab plus bevacizumab (A + T) with excellent effect to sorafenib in 2020, have enhanced the prognosis of HCC patients and increased the selectivity of treatment schemes (Kudo et al., 2018; Cheng et al., 2022). The American Gastroenterological Association (AGA) suggests for patients with preserved liver function, A + T can improve the OS of patients with sorafenib but exclude those who are not suitable for immunotherapy and/or are at a high risk of bleeding (Su et al., 2022). Compared with sorafenib, lenvatinib has promising progression-free survival (PFS), but is more prone to hypertension and skin adverse events. The A + T regimen may become the mainstream of the first-line treatment regimen for patients undergoing HCC, but sorafenib will continue to be used to become a first-line therapy for those suffering from HCC for a long period.

As the oral small molecule multityrosine kinase inhibitor (TKI) that can hinder angiogenesis, sorafenib generates an anticancer impact through hindering vascular endothelial growth factor receptor (VEGFR) and fibroblast growth factor receptor (FGFR) (Morse et al., 2019). Although sorafenib significantly prolonged the OS of patients compared with placebo, disease control rate (DCR) was only 43% and PFS approximately 4 months, indicating that more than half of patients did not respond and patients who responded developed resistance in a short time (Llovet et al., 2008; Kudo et al., 2018). In the face of the non-response and high drug resistance rate of sorafenib, active anti-tumor treatment in the back line can benefit the survival of patients. Currently, the second-line treatment approved by FDA includes cabozantinib, regorafenib, pembrolizumab and ramucirumab (HCC patients with AFP>400 ng/ml). These second-line drugs have significantly prolonged OS in HCC patients after sorafenib failure compared with placebo, whereas the lack of head-to-head clinical data limits the level of evidence for second-line treatment options. Clinicians choose second-line treatment schemes mostly based on work experience rather than experimental evidence.

In recent years, PD-1 inhibitors have benefited a variety of cancers. Even though the use of nivolumab and pembrolizumab in HCC patients has promoted the treatment of HCC patients into the era of immunity, the curative effect is not satisfactory (Finn et al., 2020b; Yau et al., 2022). However, TKI combined with PD-1 inhibitors has become the promising treatment option. In KEYNOTE-524, pembrolizumab combined with lenvatinib significantly improved the median OS (22 months) of patients with unresectable HCC(uHCC) (Finn et al., 2020a). In RESCUE, the 18-month survival rate of HCC patients reached 58.1% by camrelizumab combination with apatinib (Xu et al., 2021). Although there are no reports of randomized controlled trials of TKI combined with PD-1 inhibitors compared with TKI monotherapy as the first-line treatment for HCC, in retrospective studies, numerous studies have revealed that the combined treatment of OS and PFS is significantly better than TKI monotherapy (Li et al., 2022; Wu et al., 2022). Nevertheless, it is not clear whether TKI combined with PD-1 inhibitors is better than TKI alone in the second-line treatment. Considering the dilemma of choosing the second-line treatment, and the significant advantages of TKI combined with PD-1 inhibitors in the first-line treatment environment, it is likely to become the best choice for the second-line treatment after sorafenib failure. Additionally, this work attempted to compare the efficacy and safety of TKI monotherapy and TKI in combination with PD-1 inhibitors in HCC patients after sorafenib failure.

## Methods

### Study design and participants

This is the retrospective research carried out in the fifth medical center of the General Hospital of the Chinese people’s Liberation Army in China. From July 2018 to July 2021, HCC patients receiving TKI or TKI combined with PD-1 inhibitors as second-line treatment were included. The eligibility criteria included (1) patients diagnosed with uHCC pathologically or by two imaging techniques following the American Association for the Study of Liver Diseases (AASLD) guidelines (Marrero et al., 2018); (2) Child-Pugh class A or B; (3) an Eastern Cooperative Oncology Group (ECOG) scale performance score of 0–1; (4) tumor progression after first-line sorafenib therapy; and (5) at least one measurable tumor lesion. Besides, the exclusion criteria contained: (1) current or a history of another malignant tumor; (2) discontinued sorafenib due to the unacceptable toxicity; and (3) missing data. The approval of this study was obtained from the Chinese registered clinical trial ethics committee, and the implementation scheme was in consistence with the declaration of Helsinki in 1975. Patients are treated according to the dosage and method of TKI or PD-1 inhibitors recommended in the relevant instructions. All included patients were divided into TKI monotherapy group (TKI group) and TKI combined with PD-1 inhibitors treatment group (combination group) using different treatment methods. Demographic characteristics (including age and gender), blood indicators (including liver function, coagulation function, routine blood and tumor markers), and characteristics were collected and evaluated at baseline.

### Endpoints and follow-up

OS was the primary endpoint of this work, which referred to the time interval from initiation of treatment to death from any reason or end of the study, whichever came the first. The secondary endpoints of this work contained progression-free survival (PFS) (determined as the time from the initial dose to the first radiologically confirmed progressive disease (PD) or death from any cause), disease control rate (DCR), and objective response rate (ORR). After treatment initiation, we recorded radiological response by dynamic computed tomography (CT) or magnetic resonance imaging (MRI) at baseline and every 8–12 weeks. The Response Evaluation Criteria in Solid Tumors (RECIST) was adopted for evaluating tumor response. According to the National Cancer Institute Common Terminology Criteria for Adverse Events version 5.0, we assessed adverse events (AEs).

### Statistical analysis

Categorical data are shown to be the frequency with proportion and explored based on Chi-square test or Fisher’s exact test. With the aim of calculating the PFS and OS and plot the curve, the Kaplan-Meier method was employed. The log-rank test was adopted for comparing the two groups. A 2-tailed *p*-value ≤0.05 represented statistical significance. Cox proportional hazards models were applied, aiming to explore the correlation between the covariates and PFS or OS. Variables showing *p* < 0.05 in univariate analysis were subjected to stepwise multivariate analysis. Moreover, all data calculations were conducted by employing R language version 4.0.4 (R Foundation for Statistical Computing, Vienna, Austria).

## Results

### Patient characteristics

Totally 121 patients with unresectable HCC after failure on sorafenib were screened here from July 2018 to July 2021 in our center. Among them, we excluded 29 patients, containing 9 patients who did not receive treatment as prescribed, 7 patients who were intolerant after receiving sorafenib, 5 patients undergoing liver resection before systemic therapy, 4 patients lacking any effective follow-up, 2 patients with BCLC stage A, and 1 patient without evaluable lesions. Finally, totally 92 patients met the inclusion and exclusion criteria, including 50 in the TKI group and 42 in the combination group. The agents in TKI group included lenvatinib (*n* = 39, 78.0%), regorafenib (*n* = 8, 16.0%) and apatinib (n = 3, 6.0%). The main combination therapies included sorafenib plus sintilimab (*n* = 21, 50.0%) and lenvatinib plus camrelizumab (*n* = 6, 14.2%) (Supplementary Figure S1). At the time of data cutoff (August 2022), the median duration of follow-up was 19 (95% CI: 16.5–21.4) months. The patients were mainly male (n = 79, 85.9%). The BCLC stage of 80 (87.0%) patients was stage C at the time of enrollment. The etiology was mainly HBV(n = 85,92.4%), and there were 55 patients (60.0%) with extrahepatic metastasis. No significant difference was found in all baseline data between the sorafenib TKI group and the combination group (Table 1).

**TABLE 1:** Baseline patient characteristics.

	TKI group (n = 50), n **(%)**	Combination group (n = 42), n **(%)**	P
Age (mean ± SD)	53.4 ± 8.73	54.9 ± 8.51	0.411
Gender			0.734
Female	6 (12.0)	7 (16.7)	
Male	44 (88.0)	35 (83.3)	
Diabetes	13 (26.0)	7 (16.7)	0.408
Hypertension	16 (32.0)	8 (19.0)	0.242
Smoking	24 (48.0)	19 (45.2)	0.956
Alcohol Consumption	17 (34.0)	18 (42.9)	0.512
Chronic Liver Disease			0.698
HBV	47 (94.0)	38 (90.5)	
HCV	3 (6.0)	4 (9.5)	
Maximal Diameter			1.000
<5 cm	22 (44.0)	19 (45.2)	
≥5 cm	28 (56.0)	23 (54.8)	
PS score			1.000
0	36 (72.0)	30 (71.4)	
1	14 (28.0)	12 (28.6)	
BCLC			1.000
B	7 (14.0)	5 (11.9)	
C	43 (86.0)	37 (88.1)	
Child Pugh			0.719
A	35 (70.0)	27 (64.3)	
B (total)	15 (30.0)	15 (35.7)	
B 7	11 (22.0)	10 (23.8)	
B 8	4 (8.0)	5 (11.9)	
Macrovascular tumor thrombosis	26 (52.0)	24 (57.1)	0.777
Extrahepatic metastasis	27 (54.0)	28 (66.7)	0.307
AFP			0.098
<200	30 (60.0)	17 (40.5)	
≥200	20 (40.0)	25 (59.5)	

HBV, hepatitis B virus; HCV, hepatitis C virus; PS, performance status; BCLC, barcelona clinic liver cancer; AFP, alpha-fetoprotein.

### Efficacy

**FIGURE 1:**
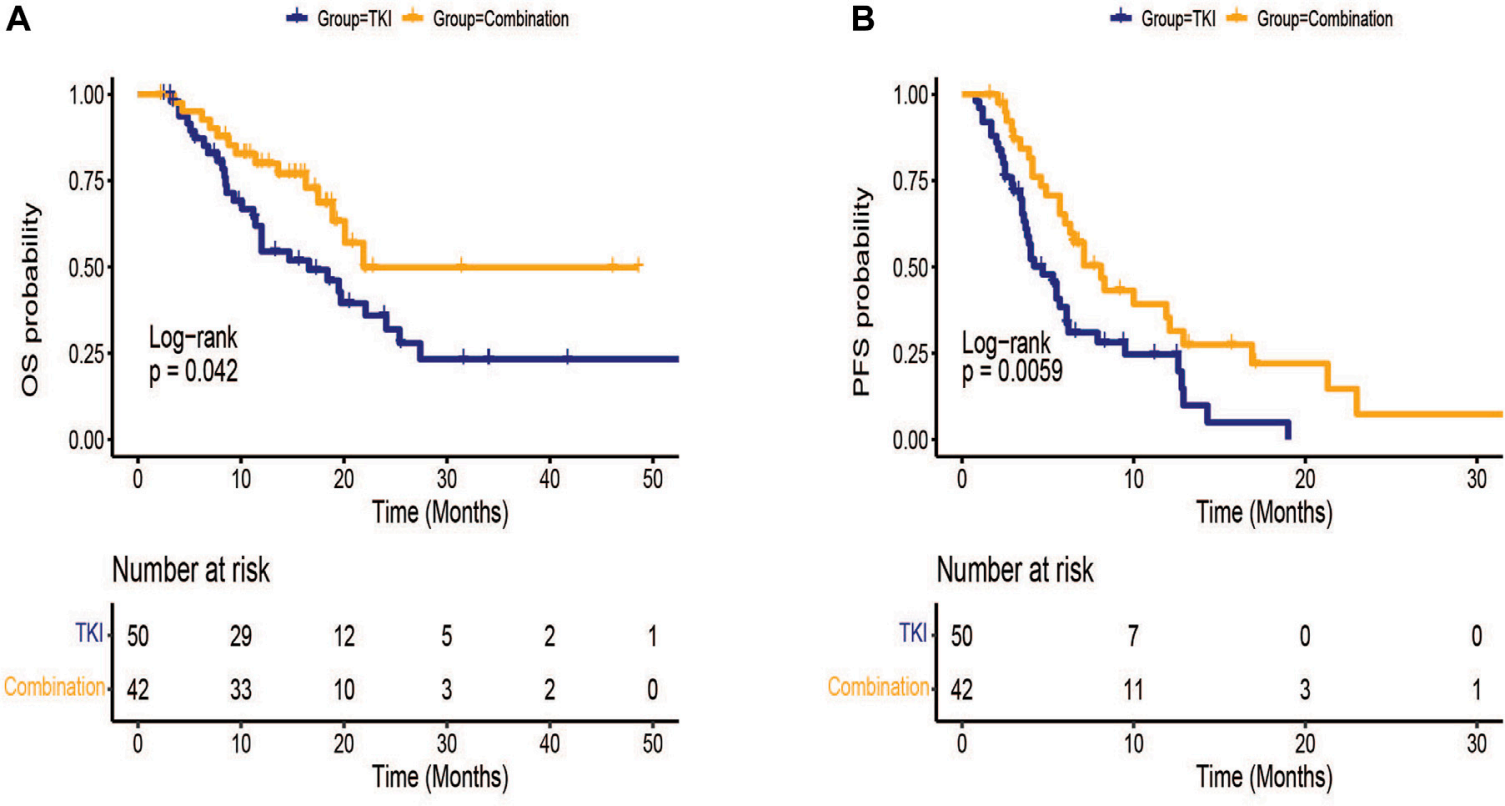
Kaplan-Meier survival curves of treatment outcome including **(A)** overall survival (OS) and **(B)** progression-free survival(PFS) between TKI group and combination group.

All patients had at least one follow-up image for radiological tumor response assessment (Table 2). It was found that the ORR rates of the combination group and TKI group were 8.0% and 9.5%, separately. The DCR of the combination group was better than TKI group (71.4% vs. 50.0%, *p* = 0.037). Efficacy in the combination group was statistically better than that in TKI group in terms of OS [median (95% CI): 21.9 (NE-NE) vs. 16.6 (10.2–23.0) months, *p* = 0.042] and PFS [median (95% CI):8.1 (5.9–10.3) vs. 4.7 (3.2–6.2) months, *p* = 0.006] (Figure 1).

**TABLE 2:** Tumor response.

	TKI group (n = 50), **n (%)**	Combination group (n = 42), **n (%)**	P
PR	4 (8.0)	4 (9.5)	
SD	21 (42.0)	26 (61.9)	
PD	25 (50.0)	12 (28.5)	
ORR	4 (8.0)	4 (9.5)	1.000
DCR	25 (50.0)	30 (71.4)	0.037
mPFS (months)	4.7	8.1	0.005
mOS (months)	16.6	21.9	0.042

PR, partial response; SD, stable disease; PD, progressive disease; ORR, objective response rate; DCR, disease control rate; mPFS, median progression free survival; mOS, median overall survival.

### Subgroup analysis

**FIGURE 2:**
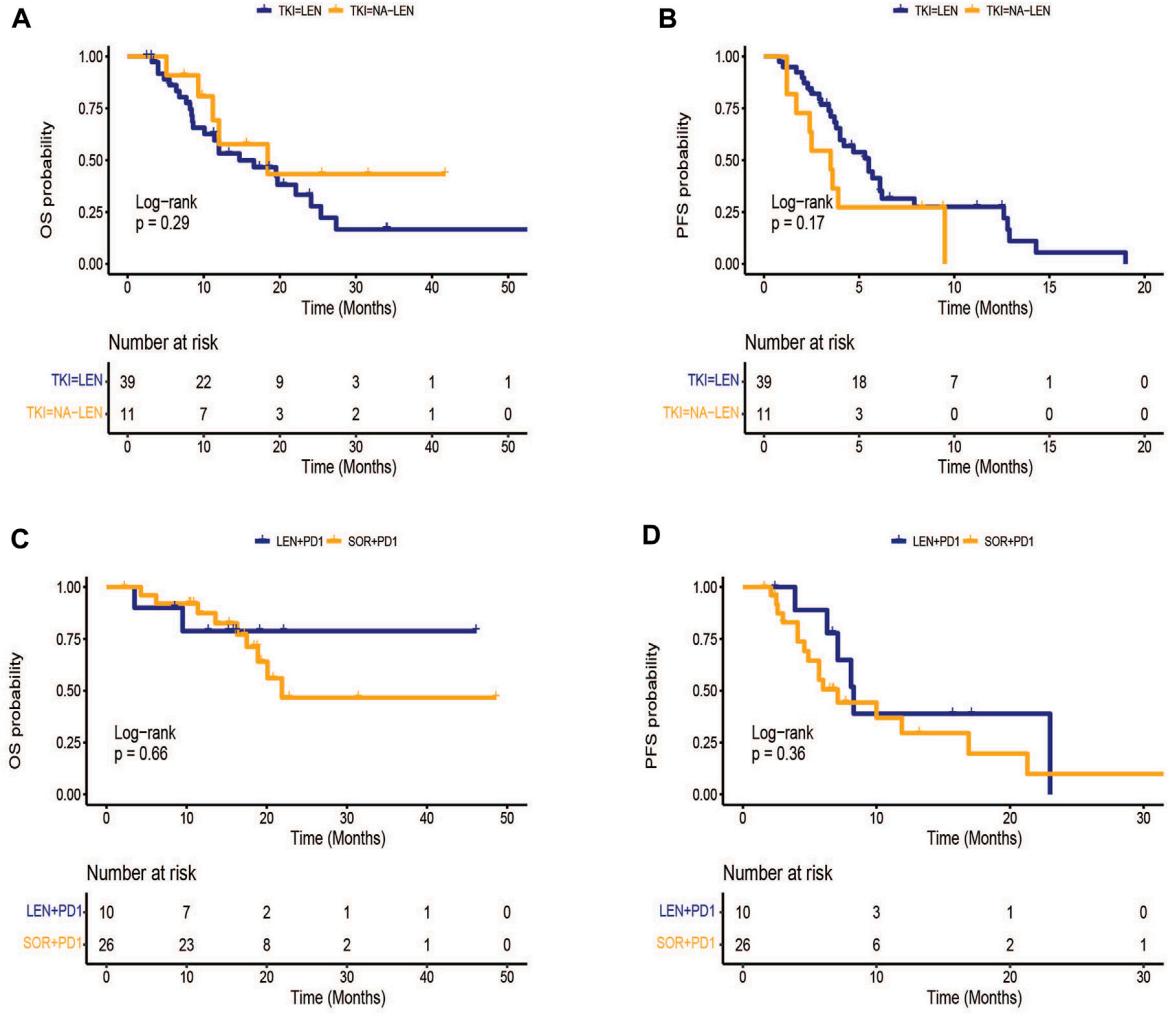
Kaplan-Meier survival curves of treatment outcome including **(A)** overall survival (OS), **(B)** progression-free survival(PFS) between lenvatinib and other TKI groups, **(C)** OS, **(D)** PFS between sorafenib plus PD-1 inhibitors and lenvatinib plus PD-1 inhibitors.

The patients in the TKI group were classified into lenvatinib (*n* = 39) and other TKI(*n* = 11) groups. The median OS was 14.7 (95% CI: 6.7–22.7) months in lenvatinib group, while the 13 months survival rate in the control group was 18.4%(95% CI: 3.2–33.6) (*p* = 0.291)(Figure 2A).The median PFS was 5.5 (95% CI: 3.8–7.2) months in lenvatinib group, while the control group was 3.5 (95% CI: 2.2–4.7) months (*p* = 0.174) (Figure 2B). In the subgroup analysis in the combination group, there existed no obvious difference in median PFS (8.3 vs. 7.1 months, *p* = 0.364) and median OS (NE vs. 21.9 months, *p* = 0.657) between sorafenib in combination with PD-1 inhibitors and lenvatinib in combination with PD-1 inhibitors (Figures 2C,D).

### Factors influencing efficacy

Table 3 shows the factors associated with the patient’s PFS and OS. In univariate analysis, TKI monotherapy, ECOG-PS score 1, and maximum tumor diameter greater than 5 cm were independently related to a shortened PFS, while TKI monotherapy and ECOG-PS score 1 were independently associated for shortened OS. In multivariate analysis, the independently correlated with a shortened PFS included, ECOG-PS score 1 (HR 1.8, 95% CI: 1.1–3.0, *p* = 0.027) and tumor diameter greater than 5 cm (HR 1.8, 95% CI: 1.1–2.9, *p* = 0.028), whereas the independently associated with a shortened OS was only ECOG-PS score 1 (HR 1.9, 95% CI: 1.0–3.6, *p* = 0.021). The combination group had better PFS (HR 0.5, 95% CI: 0.3–0.8, *p* = 0.005) and prolonged OS (HR 0.5, 95% CI: 0.3–1.0, p = 0.051) compared to the TKI group.

**TABLE 3:** Analysis of prognostic risk factors.

	Progression-free survival			Overall survival		
	HR	95% CI	P	HR	95% CI	P
Univariate analysis						
Age>60, yeares	0.9	0.4–1.9	0.700	0.4	0.1–1.4	0.160
TKI group	0.5	0.3–0.8	0.007	0.5	0.3–1.0	0.045
Male sex	1.2	0.6–2.3	0.650	1.3	0.5–3.0	0.600
Diabetes	1.3	0.7–2.5	0.370	1.0	0.5–2.0	0.930
Hypertension	1.3	0.8–2.3	0.310	1.9	0.8–4.5	0.150
Smoking	1.0	0.6–1.7	0.900	1.1	0.6–1.9	0.860
Alcohol-Consumption	1.1	0.7–1.8	0.650	0.9	0.5–1.7	0.810
HCV	1.1	0.4–2.7	0.850	1.3	0.5–3.5	0.670
PS score 1	1.9	1.1–3.2	0.015	2.1	1.1–3.9	0.018
Largest tumor size ≥5 cm	1.7	1.0–2.8	0.040	1.2	0.7–2.3	0.480
BCLC (C)	1.1	0.6–2.3	0.710	0.9	0.4–2.1	0.800
Child-Pugh	1.6	0.9–2.6	0.100	1.0	0.5–1.9	0.940
Macrovascular tumor thrombosis	1.3	0.8–2.2	0.270	1.3	0.7–2.3	0.440
Extrahepatic metastasis	0.8	0.5–1.4	0.450	0.8	0.5–1.5	0.570
AFP>=200 ng/ml	1.1	0.7–1.7	0.820	0.9	0.5–1.5	0.590
Multivariate analysis						
Combination group	0.5	0.3–0.8	0.005	0.5	0.3–1.0	0.051
PS score 1	1.8	1.1–3.0	0.027	2.1	1.1–3.8	0.021
Largest tumor size ≥5 cm	1.8	1.1–2.9	0.028			

HR, hazard ratio; CI, confidence interval; HCV, hepatitis C virus; PS, performance status; BCLC, barcelona clinic liver cancer; AFP, alpha-fetoprotein.

### Safety

The incidence rates of grade ≥1 adverse event in the TKI group and the combination group were 96.0% and 97.6%, respectively. Obviously, the most common adverse event in the TKI group was neutropenia (*n* = 24,48.0%) and hypoalbuminemia (*n* = 23, 54.8%) in the combination group. In addition, the common grade 3–4 adverse events in the TKI group were leukopenia (*n* = 6, 12.0%), thrombocytopenia (*n* = 6, 12.0%), hypertension (*n* = 3, 6.0%), and lymphopenia (*n* = 3, 6.0%). The common grade 3–4 adverse events in the combined group included lymphopenia (*n* = 9, 21.4%), leukopenia (*n* = 3, 9.5%), hypertension (*n* = 2, 4.7%), and thrombocytopenia (*n* = 2, 4.7%). In the TKI group, the agent dose was decreased in 1 case due to grade 3 hypertension. In the combination group, two patients discontinued immunotherapy, including 1 with immune-related pneumonitis and 1 with immune-related myocarditis. No patients died due to adverse events (Table 4).

**TABLE 4:** Treatment-related adverse events (TRAEs).

Adverse event	TKI group (n = 50)		Combination group (n = 42)	
	Grade 1–2	Grade 3–4	Grade 1–2	Grade 3–4
Any treatment-related adverse event, n (%)	48 (96.0)	15 (30.0)	41 (97.6)	16 (38.0)
Diarrhea, n (%)	12 (24.0)	0	7 (16.7)	1 (2.3)
Fatigue, n (%)	11 (22.0)	0	6 (14.2)	0
Hand and foot syndrome, n (%)	6 (12.0)	2 (4.0)	4 (9.5)	1 (2.3)
Hypertension, n (%)	4 (8.0)	3 (6.0)	5 (11.8)	2 (4.7)
Decreased appetite, n (%)	5 (10.0)	0	3 (7.1)	0
Proteinuria, n (%)	5 (10.0)	0	1 (2.3)	0
Hypothyroidism, n (%)	4 (8.0)	0	1 (2.3)	0
Rash, n (%)	2 (4.0)	0	3 (7.0)	0
Immune-related pneumonia, n (%)	0	0	1 (2.3)	0
Myocarditis, n (%)	0	0	1 (2.3)	0
Laboratory test, n (%)				
Neutropenia, n (%)	24 (48.0)	1 (2.0)	11 (26.2)	1 (2.3)
Leukopenia, n (%)	21 (42.0)	6 (12.0)	13 (30.8)	3 (9.5)
Hypoalbuminemia, n (%)	21 (42.0)	0	23 (54.8)	0
Fibrinogen decreased, n (%)	19 (38.0)	0	13 (30.8)	0
Lymphopenia, n (%)	16 (32.0)	3 (6.0)	8 (19.0)	9 (21.4)
Alanine aminotransferase increased, n (%)	16 (32.0)	1 (2.0)	5 (11.9)	0
Thrombocytopenia, n (%)	15 (30.0)	6 (12.0)	12 (28.5)	2 (4.7)
Blood lactate dehydrogenase increased, n (%)	13 (26.0)	0	10 (23.8)	0
Aspartate aminotransferase increased, n (%)	12 (24.0)	1 (2.0)	7 (16.7)	0
Hypocalcemia, n (%)	10 (20.0)	0	13 (31.0)	0
Hypokalemia, n (%)	10 (20.0)	0	14 (33.4)	0
Anemia, n (%)	8 (16.0)	1 (2.0)	12 (28.5)	0
Hypophosphatemia, n (%)	8 (16.0)	0	13 (31.0)	0
Hyperuricemia, n (%)	5 (10.0)	0	4 (9.5)	0
Serum amylase increased, n (%)	4 (8.0)	0	4 (9.6)	0
Creatinine increased, n (%)	0	0	3 (7.1)	0

## Discussion

It is acknowledged that this is the first retrospective cohort study comparing treatment response and adverse events between TKI alone and TKI combined with PD-1 inhibitors as a second-line for uHCC. Our findings showed that combination therapy may improve DCR, PFS, and OS in patients in comparison with TKI monotherapy. There existed no statistically significant difference in adverse events between the two groups.

In the IMbrave 150 study, the PFS (6.9 months vs. 4.3 months, *p* < 0.001) and OS (19.2 months vs. 13.4 months, *p* < 0.001) of the A + T regimen were significantly prolonged compared with sorafenib monotherapy, and thus the regimen was recommended by the FDA as the standard first-line treatment regimen for uHCC patients (Cheng et al., 2022). The success of this combination therapy has brought novel hope to patients, and the synergistic effect of anti-vascular drugs combined with immune checkpoint inhibitors has already become the focus of patients and doctors. In prospective studies, TKI combined with PD-1 inhibitors (including pembrolizumab plus lenvatinib and camrelizumab plus apatinib) had a promising OS [(Finn et al., 2020a; Xu et al., 2021)]. In the real world, TKI combined with PD-1 inhibitors therapy has obviously prolonged OS in comparison with TKI monotherapy, including lenvatinib plus nivolumab vs. lenvatinib monotherapy (22.9 months vs. 10.3 months, *p* = 0.01) (Wu et al., 2022), lenvatinib plus camrelizumab vs. lenvatinib monotherapy (not reached vs. 13.9 months, *p* = 0.02) (Li et al., 2022), and lenvatinib plus sintilimab vs. lenvatinib monotherapy (21.7 months vs. 12.8 months, *p* = 0.01) (Zhao et al., 2022). Based on the above study, lenvatinib combined with PD-1 inhibitors had a significantly prolonged OS in first-line treatment of uHCC compared with lenvatinib monotherapy. The above studies showed that lenvatinib combined with PD-1 inhibitors significantly prolonged OS in first-line treatment of uHCC compared with lenvatinib monotherapy. In this work, although the TKI of the combination regimen was mainly sorafenib (61.9%) and was used in the second-line treatment of uHCC, the combination regimen also had a better prognosis than TKI monotherapy.

Sorafenib significantly prolongs OS compared to placebo and is widely used worldwide as first-line therapy in uHCC patients (Llovet et al., 2008). Unfortunately, a large number of HCC patients show a poor response to sorafenib or exhibit resistance to sorafenib treatment within 6 months (Chen et al., 2015). Continuing systemic therapy after sorafenib failure is the most effective way to prolong OS. In RESORCE, in patients undergoing HCC who failed sorafenib, continued regorafenib treatment significantly prolonged OS compared with placebo (10.6 months vs. 7.8 months, *p* < 0.001). The median time to death remained longer in the regorafenib group when survival was evaluated from prior sorafenib (vs. placebo, 26.0 months vs. 19.2 months) (Finn et al., 2018). The benefit of regorafenib for patients after failure of sorafenib was further confirmed in several retrospective clinical studies (Granito et al., 2021a). Recently, many second-line treatment studies have been carried out for HCC patients after sorafenib failure. FDA-approved second-line therapy-targeted drugs that have shown survival benefits in phase 3 clinical trials, including regorafenib (mOS, 10.6 months) (Finn et al., 2018), cabozantinib (mOS, 10.2 months) (Abou-Alfa et al., 2018) and ramucirumab (mOS, 8.5 months) (Zhu et al., 2019). Approved second-line immune monotherapy include nivolumab (mOS, 15.6 months) (El-Khoueiry et al., 2018) and pembrolizumab (mOS, 13.8 months) (Finn et al., 2020b). Additionally, the combination regimen nivolumab plus ipilimumab has not completed a phase 3 clinical trial, but has received FDA accelerated approval in a second-line setting due to long OS (mOS, 22.8 months) (Yau et al., 2020). Furthermore, second-line combination therapy options that are expected to be approved are durvalumab plus tremelimumab (mOS, 18.7 months) (Kelley et al., 2021) and camrelizumab plus apatinib (18 months OS rates, 56.5%) (Xu et al., 2021). Moreover, many second-line drugs that have completed phase 2 clinical trials or have been approved in some countries are booming, including apatinib (mOS, 8.7 months) (Qin et al., 2021), tislelizumab (mOS, 12.4 months) (Ducreux et al., 2021) and camrelizumab (mOS, 13.8 months) (Qin et al., 2020).

Faced with so many second-line treatment options, how to determine the best treatment has become the most perplexing problem. To determine the best second-line treatment regimen, we performed the analysis from different perspectives. First, based on the prospective second-line studies, the combination therapy regimen has a better OS than the monotherapy (targeted therapy or immunotherapy). Nevertheless, such conclusions need to be cautious, because some of the above studies have only completed the phase 2 clinical trials, even if phase 3 clinical trials have been completed but only use placebo as a control. Second, in the real world, controlled trials of second-line drugs only compared single agents and did not screen for superiority, including regorafenib *versus* nivolumab (Choi et al., 2020), regorafenib *versus* cabozantinib (Casadei-Gardini et al., 2021) and cabozantinib *versus* ramucirumab (Trojan et al., 2021). Our results suggest that there is a marginal difference in PFS with lenvatinib compared with other TKI agents (5.5 vs. 3.5 months, *p* = 0.147). Previously, lenvatinib is superior to sorafenib of PFS in both prospective and retrospective studies (Kudo et al., 2018; Kuo et al., 2021), and thus it may be preferentially recommended in patients who cannot use immunotherapy after sorafenib failure. Certainly, for patients who can use immunotherapy, TKI combined with PD-1 inhibitors as a second-line regimen is a good option in line with our results. Third, the same treatment may exert different effects in different countries or regions. The primary risk factor for non-Japanese Asian patients is HBV, while European and American patients are HCV (El-Serag, 2012). The median OS of HCC patients treated with sorafenib was 10.7 months in Europe, Australasia and the United States, and 6.5 months in China, Taiwan, and South Korea (Llovet et al., 2008; Cheng et al., 2009). Due to the differences in regions and etiologies, although apatinib has been approved to be the second-line treatment in China, the efficacy of this regimen in other countries needs further investigation since the phase 3 clinical trial only included Chinese patients (Qin et al., 2021). Fourth, the current studies on second-line therapy choices for HCC patients are all conducted with sorafenib as a first-line treatment. Intolerance or disease progression due to sorafenib is related to response to second-line therapy. Ramucirumab and pembrolizumab were effective for patients with disease progression after sorafenib treatment, but not for the intolerant to sorafenib (Zhu et al., 2019; Finn et al., 2020b). In RESORCE, patients who were intolerant to sorafenib were excluded from the enrolled patients receiving second-line regorafenib excluded. However, cabozantinib can bring benefits after sorafenib treatment in patients with disease progression or intolerance, and thus it is the only second-line TKI recommended by the AGA for use in patients with sorafenib intolerance (Kelley et al., 2020; Su et al., 2022). Our study also excluded sorafenib-intolerant patients. Therefore, the efficacy of TKI combined with PD-1 inhibitors in sorafenib-intolerant patients in the second-line setting needs to be further explored in follow-up studies.

A comprehensive analysis of the above-mentioned second-line treatment decision-making perspectives, combined with our findings, shows that there is potential value in recommending combination therapy after sorafenib failure. (1) At present, the commonly used second-line single drugs are TKI drugs, and thus there may be cross-resistance with sorafenib which can greatly limit the survival of patients. (2) Commonly used TKI drugs all exert the targeted therapeutic effect of VEGFR, which can not only regulate tumor blood vessels, but also serve as an effective immunomodulatory molecule, affecting TAM, MDSC, Treg cells and effector T cells (Fukumura et al., 2018). Nevertheless, PD-1 inhibitors can restore effector CD8^+^ T cell function by blocking extensive dephosphorylation between PD-L1 and PD-1, which can impair or abolish the immunosuppressive effects caused by Treg cells and ultimately inhibit tumor growth (Ahn et al., 2018; Granito et al., 2021b). Multiple mouse experiments have demonstrated that TKI combined with PD-1 inhibitors combination therapy can achieve the synergistic effect (Sprinzl et al., 2015; Torrens et al., 2021). (3) Multiple studies have revealed that the toxicity profile and tolerance were similar between TKI monotherapy and combination regimens (Li et al., 2022; Wu et al., 2022). (4) There are many combinations of TKI combined with PD-1 inhibitors, which can avoid the limitations of a certain drug and increase the practicality of the treatment plan. Our subgroup analysis proved that there existed no obvious difference in OS and PFS between sorafeinib combined with PD-1 inhibitors and lenvatinib combined with PD-1 inhibitors, which could also increase the possibility that different combinations may benefit from. However, the mechanism of lenvatinib and sorafenib combined with PD-1 inhibitors is different, the former can specifically reduce the abundance of tumor Treg cells (Torrens et al., 2021), while the latter has the effect of directly inhibiting the activation of M2 macrophages (Sprinzl et al., 2015). The effect of this combination treatment is promising. However, follow-up large-sample and prospective studies need to be performed to explore what kind of combination is more effective and what kind of situation is used.

Several limitations have to be mentioned in this study. First, this study was designed as a retrospective one with the small sample size, which could generate information bias and selection bias. Moreover, we explored multiple second-line TKI or PD-1 inhibitors. The clinical efficacy of specific regimens must be explored in future clinical trials. Third, our study excluded patients with sorafenib intolerance. Thus, the efficacy of TKI in combination with PD-1 inhibitors was not available in these patients.

## Conclusion

To conclude, TKI combined with PD-1 inhibitors may benefit more than TKI monotherapy in HCC patients after sorafenib failure. Prospective studies with large samples are required to explore and clarify specific treatment options for patients.

## Data Availability

The original contributions presented in the study are included in the article/Supplementary Material, further inquiries can be directed to the corresponding authors.
