# Supplementary figures and images for: TKI or TKI combined with PD-1 inhibitors as second-line treatment for HCC patients after sorafenib failure
Source: Front Pharmacol. 2022 Dec 9;13:1026337. doi: 10.3389/fphar.2022.1026337 (PMC9782409; doi:10.3389/fphar.2022.1026337)

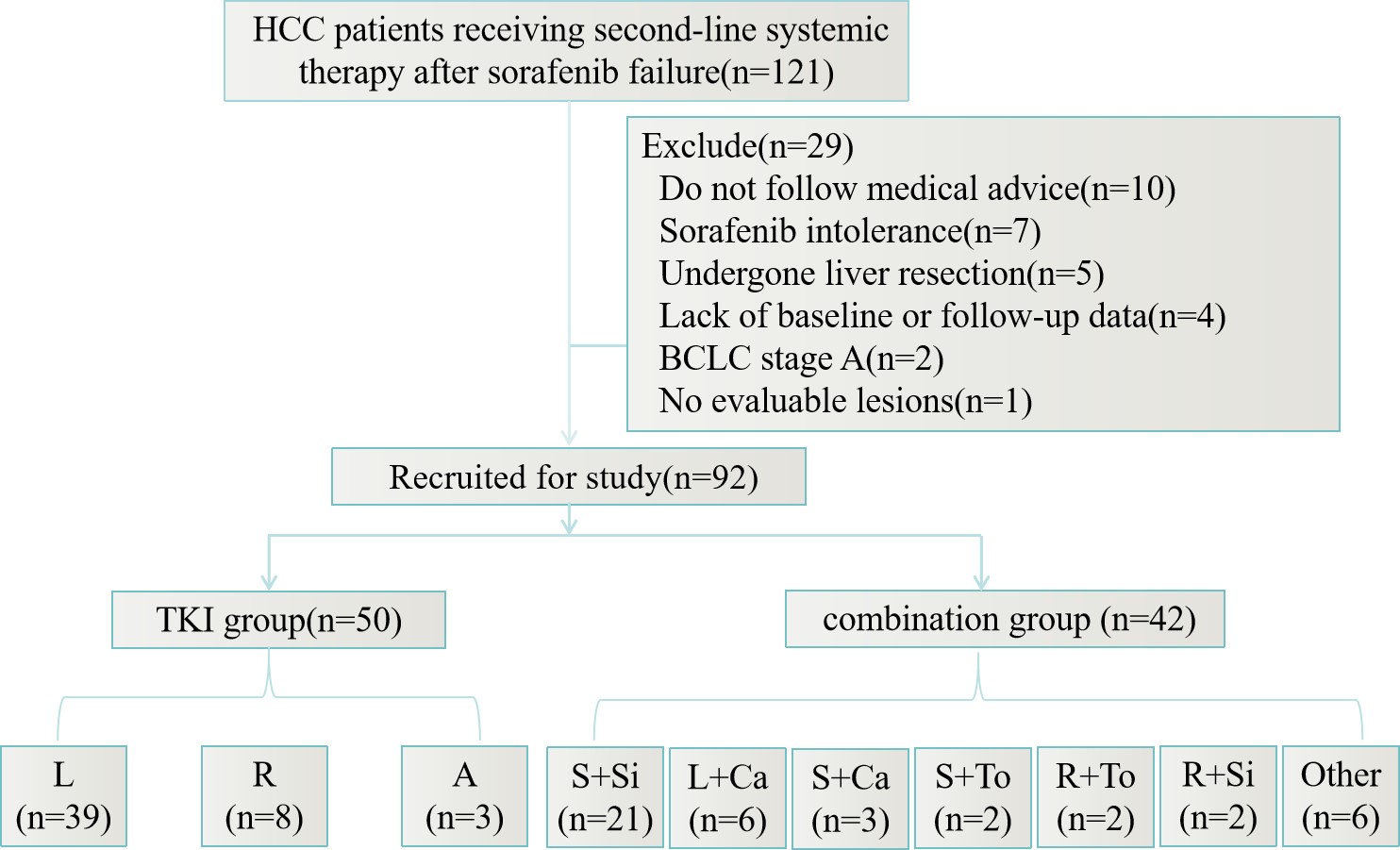

Supplement: Supplementary file 1 [file Image1.jpeg]
